# The protective effects of a phosphodiesterase 5 inhibitor, sildenafil, on postresuscitation cardiac dysfunction of cardiac arrest: metabolic evidence from microdialysis

**DOI:** 10.1186/s13054-014-0641-7

**Published:** 2014-12-05

**Authors:** Qian Zhang, Wei Yuan, Guoxing Wang, Junyuan Wu, Miaomiao Wang, ChunSheng Li

**Affiliations:** Department of Emergency Medicine, Beijing Chao-yang Hospital, Capital Medical University, 8# Worker’s Stadium South Road, Chao-yang District, Beijing, 100020 China; Department of Emergency Medicine, Beijing You-yi Hospital, Capital Medical University, 95# Yong-an Road, Xuan-wu District, Beijing, 100050 China

## Abstract

**Introduction:**

Recent experimental and clinical studies have indicated the cardioprotective role of sildenafil during ischemia/reperfusion injury. The aim of this study was to determine, by obtaining metabolic evidence from microdialysis, if sildenafil could reduce the severity of postresuscitation myocardial dysfunction and lead to cardioprotection through beneficial effects on energy metabolism.

**Methods:**

Twenty-four male piglets were randomly divided into three groups: sildenafil (*n* = 8), saline (SA; *n* = 8) and sham operation (*n* = 8). Sildenafil pretreatment consisted of 0.5 mg/kg sildenafil administered once intraperitoneally 30 minutes prior to ventricular fibrillation (VF). The myocardial interstitial fluid (ISF) concentrations of glucose, lactate, pyruvate, glutamate and glycerol were determined by microdialysis before VF. Afterward, the piglets were subjected to 8 minutes of untreated VF followed by 15 minutes of open-chest cardiopulmonary resuscitation. ISF was collected continuously, and the experiment was terminated 24 hours after resuscitation.

**Results:**

After 8 minutes of untreated VF, the sildenafil group exhibited higher glucose and pyruvate concentrations of ISF and lower lactate and glutamate levels in comparison with the SA group, and these data reached statistical significance (*P* < 0.05). Advanced cardiac life support was delivered to both groups, with a 24-hour survival rate showing a promising trend in the sildenafil group (7 of 8 versus 3 of 8 survivors, *P* < 0.05). Compared with the SA group, the sildenafil group had a better outcome in terms of hemodynamic and oxygen metabolism parameters (*P* < 0.05). Myocardial tissue analysis revealed a dramatic increase in the contents of ATP, ADP and phosphocreatine in the sildenafil group versus the SA group at 24 hours after return of spontaneous circulation (ROSC; *P* = 0.03, *P* = 0.02 and *P* = 0.02, respectively). Furthermore, 24 hours after ROSC, the sildenafil group had marked elevations in activity of left ventricular Na^+^-K^+^-ATPase and Ca^2+^-ATPase compared with the SA group (*P* = 0.03, *P* = 0.04, respectively).

**Conclusions:**

Sildenafil could reduce the severity of postresuscitation myocardial dysfunction, and it produced better clearance of metabolic waste in the ISF. This work might provide insights into the development of a novel strategy to treat postresuscitation myocardial dysfunction.

**Electronic supplementary material:**

The online version of this article (doi:10.1186/s13054-014-0641-7) contains supplementary material, which is available to authorized users.

## Introduction

Morbidity and mortality due to cardiac arrest (CA) remains unacceptably high, yet effective treatments for CA have proven to be elusive [1]. In patients who initially achieve return of spontaneous circulation (ROSC) after CA, the significant subsequent morbidity and mortality are largely due to the myocardial dysfunction that accompanies prolonged whole-body ischemia. Furthermore, CA contributes to hemodynamic disorders that cause the systemic release of massive oxygen free radicals, lactic acid and metabolites of arachidonic acid, which could reach the different tissues via blood circulation and could cause ischemia/reperfusion (I/R) injury [2]. Postresuscitation myocardial dysfunction, an important component of the “post–cardiac arrest syndrome,” is caused by I/R injury and includes primary manifestations such as arrhythmias, myocyte apoptosis and contractile dysfunction [3]. In addition, myocardial dysfunction aggravates persistent precipitating pathologies, such as microcirculatory dysfunction, requiring lifelong medication and clinical follow-up.

Nitric oxide (NO) has been identified as an important mediator of the physiological and pathological processes in I/R injury [4]. Sildenafil is a selective inhibitor of the isoform 5 of the enzyme phosphodiesterase (PDE5), which is responsible for the breakdown of 39,59-cyclic guanosine monophosphate (cGMP) in smooth muscle cells [5]. As the intracellular level of cGMP is controlled by the activity of PDE5, it is expected that pharmacological inhibition of PDE5 by sildenafil could improve cardioprotection in the myocardium. As a pharmacological stimulator of ischemic preconditioning, sildenafil now represents a powerful therapeutic tool for treating several cardiovascular disorders and provides direct cardioprotection against ischemia through NO-dependent pathways [5,6]. Well-tolerated for long-term treatment with few side effects, sildenafil reduces pulmonary vascular resistance, improves arterial oxygenation in patients with pulmonary artery hypertension and prevents altitude-induced hypoxemia [7,8]; however, the potential role of sildenafil as a novel pharmacologic adjunct to resuscitation from CA for the purpose of attenuating the myocardial dysfunction caused by I/R injury remains unexplored.

The microdialysis technique is capable of detecting real-time metabolic changes *in vivo*, which makes it a sensitive and site-specific method for monitoring metabolism in the myocardial interstitial fluid (ISF) during CA [9]. With this background in mind, we designed this study to investigate changes of intracardial metabolism measured by microdialysis under different management methods in an established porcine model of CA, and we also sought to determine whether administration of sildenafil is optimal for attenuating postresuscitation myocardial dysfunction by obtaining extracellular metabolic evidence.

## Material and methods

### Ethics statement

This study was carried out in strict accordance with the guidelines for animal care and use established by the Capital Medical University Animal Care and Use Committee. The study’s experimental protocol was approved by the Committee on the Ethics of Animal Experiments of Capital Medical University (permit number 2010-D-013). The animals used in this study were handled in compliance with the Guiding Principles for the Care and Use of Animals expressed in the Declaration of Helsinki [10]. All animals were maintained in a specific pathogen-free environment in our facility and were fed standard chow and had free access to water. All surgeries were performed while the animals were under anesthesia and analgesia, and all efforts were made to minimize the animals’ suffering.

### Animal preparation

Twenty-four inbred male Wuzhishan (WZS) miniature piglets ages 11 to 13 months with an average weight of 30 ± 2 kg were used in each part of this study. Our choice of the WZS miniature piglets was based on their characteristics similar to humans with regard to histologic structures and physiology, and especially due to the highest inbreeding coefficient (>0.965) [11]. The piglets were randomly assigned to one of three groups: a sildenafil group (*n* = 8), a saline (SA) group (*n* = 8) and sham operation (SHAM) group (*n* = 8). Sildenafil was obtained from a 25-mg tablet (Pfizer, West Ryde, Australia) that was dissolved in 50 ml of saline, filtered and stored at 4°C. This solution was delivered once intraperitoneally in a dose of 0.5 mg/kg 30 minutes prior to ventricular fibrillation (VF) [12]. The drugs were delivered in a randomized manner by using the sealed envelope method. The vehicle (0.9% NaCl) was administered in the same manner and volume. The investigator was blinded to the treatment. After premedication with 0.5 mg/kg intramuscular midazolam, anesthesia was induced by ear vein injection of propofol (1.0 mg/kg) and maintained in a surgical plane of anesthesia with intravenous infusion of sodium pentobarbital (8 mg/kg/hr). All animals were intubated with a cuffed 6.5-mm endotracheal tube and ventilated with a volume-controlled ventilator (Servo 900C; Siemens, Munich, Germany) using a tidal volume of 8 ml/kg and a respiratory frequency of 12 breaths/min on room air. End-tidal partial pressure of carbon dioxide (pCO_2_) was measured by in-line infrared capnograph (CO_2_SMO plus capnograph and pulse oximeter monitor; Respironics, Murrysville, PA, USA). Respiratory frequency was adjusted to maintain end-tidal pCO_2_ between 35 and 40 mmHg before inducing CA. Room temperature was adjusted to 26°C, and body temperature was maintained at 37°C under an infrared lamp. All efforts were made to minimize the animals’ suffering. Fluid losses were compensated for by infusing 30 ml/kg of acetated Ringer’s solution during the first hour of preparation, followed by a continuous infusion of 2.5% glucose electrolyte solution at 8 ml/kg/hr and acetated Ringer’s solution 20 ml/kg/hr.

An angiographic catheter was inserted from the femoral artery into the aortic arch for reference blood samples and for measuring aortic pressure. A Swan-Ganz catheter (7-French; Edwards Lifesciences, Irvine, CA, USA) was advanced from the right femoral vein and flow-directed into the pulmonary artery for measurement of right atrial pressure, mean pulmonary arterial pressure (MPAP) and cardiac output (CO). The electrocardiograms (ECGs) and all hemodynamic parameters were monitored with a patient monitoring system (M1165; Hewlett-Packard, Palo Alto, CA, USA).

### Experimental protocol

**Figure 1:**
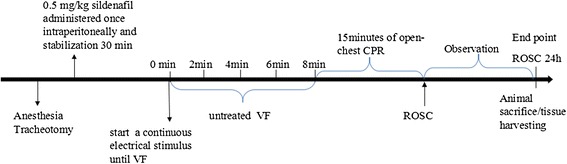
**Diagram of protocol of the first ventricular fibrillation.** Ventricular fibrillation (VF) was induced by a temporary pacemaker conductor connected to an electrical stimulator programmed in the S_1_/S_2_ mode (300/200 ms). CPR, Cardiopulmonary resuscitation; ROSC, Restoration of spontaneous circulation.

The experimental procedure of cardiopulmonary resuscitation (CPR) was performed as follows (Figure 1). After establishment of vascular catheter placement, the animals were allowed to equilibrate for 30 minutes to achieve a stable resting level. Baseline measurements of arterial blood gases were obtained. Mechanical ventilation was established as described above. The temporary pacemaker conductor was inserted into the right ventricle through the right internal jugular vein and connected to an electrical stimulator (GY-600A; Kaifeng Henan Equipment Co, Kaifeng, China) programmed in the S_1_/S_2_ mode (300/200 ms), 40 V, 8:1 proportion and 10-ms step length to provide a continuous electrical stimulus until VF was achieved [13].

**Figure 2:**
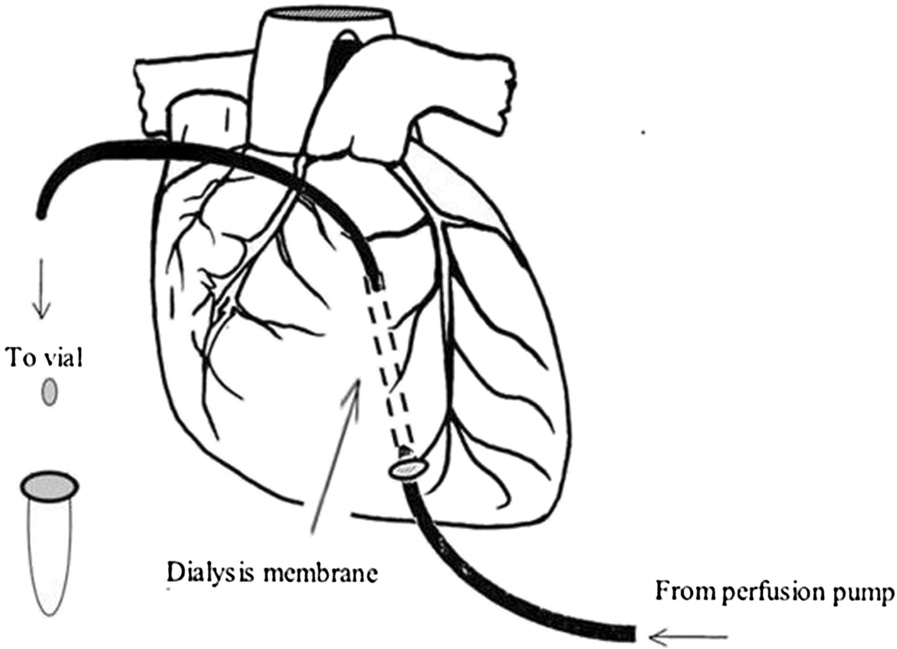
**Schematic representation of a microdialysis probe implanted into the piglet myocardium.**

The chest was opened by median strenotomy, and the heart was suspended in a pericardial cradle. Two pairs of 2-mm piezoelectric transducers were placed on the endocardial surface of the left ventricle (LV) on the major and minor axes. A tourniquet was placed around the inferior vena cava. Heparin sodium (200 U/kg) was administered intravenously before probe implantation to prevent blood coagulation [14]. Two microdialysis catheters (CMA70; CMA Microdialysis AB, Kista, Sweden) were implanted separately into the lateral wall of the LV myocardium midway between the apex and base of the heart. The CMA70 catheters were perfused with Ringer’s solution *in situ* for 45 minutes before baseline measurements were taken, and a constant flow was maintained (2.5 μl/min) using a microdialysis pump (CMA106; Microdialysis AB) [14] (Figure 2). The dialysate was collected by using a dialysate collector (CMA142; Microdialysis AB) every 30 minutes. The ISF from the LV wall was collected through the microdialysis tubes for 10 minutes (baseline). VF was then electrically induced and continued for 8 minutes. VF was defined as an electrocardiogram showing waveforms corresponding to VF and a rapid decline in mean aortic pressure (MAP) toward zero. Ventilation was stopped during VF induction, and ventilation was withheld for the entire 8-minute duration of VF. ISF was collected continuously at 0 to 2, 2 to 4, 4 to 6, and 6 to 8 minutes of VF.

After 8 minutes of untreated VF, open-chest CPR was started with a compression rate of 60 to 80 times per minute, because it is almost impossible to have a compression rate higher than 80 per minute without damaging the myocardium while handling the heart. During CPR, 100% oxygen was delivered with the same baseline ventilator settings. After 2 minutes of open-chest CPR, an internal monophasic countershock was delivered at the energy level of 20 J (Physio-Control, Redmond, WA, USA). Subsequent defibrillatory shocks were increased to 40 J, and all subsequent attempts were made at the 40-J dose [15]. If ROSC was not achieved after six shocks, a bolus of 0.4 U/kg vasopressin was administered through the right atrial catheter. CPR was continued for up to 15 minutes. CPR was discontinued if ROSC was not achieved during this period.

ROSC was defined as 10 consecutive minutes of maintenance of systolic blood pressure at 50 mmHg. If spontaneous circulation was not restored within 30 minutes, we regarded the animal as dead [16]. All the animals received normal saline (10 ml/kg/hr) intraoperatively to replenish fluid losses. Five minutes after ROSC, oxygen was reset to 30%. Minute ventilation and respiratory frequency were also maintained accordingly to maintain end-tidal pCO_2_ and were adjusted to maintain an arterial PaCO_2_ within the range of 5.0 kPa to 5.5 kPa if arterial pH was <7.20. If the base deficit was more than 10 mmol/L 5 minutes after ROSC, acidosis was corrected with a 1 mmol/kg Tris buffer mixture (Tribonat; Kabi Fresenius, Stockholm, Sweden) and by increasing minute ventilation [16]. The animals were continuously anesthetized and underwent a 24-hour intensive care period. With the exception of a right internal jugular vein sheath that was used for fluid administration, all other vascular sheaths were removed 6 hours after ROSC. The anesthetized animals were constantly monitored and underwent a 24-hour intensive care period when Ringer’s solution (20 ml/kg/hr) was administered. Next, the anesthetized piglets were killed with 10 ml of 10 mol/L potassium chloride intravenously following a bolus of 100 mg of propofol intravenously 24 hours after ROSC, when the last measurements were made. Myocardial specimens were snap-frozen in liquid nitrogen and stored at −80°C.

### Measurements

#### Hemodynamic and oxygen metabolism parameters collection

ECGs were continuously monitored. The hemodynamic parameters, including heart rate (HR), CO, MAP and MPAP, were measured continuously. We recorded the values at baseline, 30 minutes and 1, 2, 4 and 6 hours after ROSC. At the end of each time point, 4°C saline was injected into the right atrium through the Swan-Ganz catheter to determine CO by the transpulmonary thermodilution method as described previously [16]. MAP was determined by the electronic integration of the aortic blood pressure waveform. The amounts of infused fluid and urine output were also monitored during the experiment. Serum lactate levels and arterial blood gas values for which temperatures were corrected to 37°C were measured regularly using an ABL 520 Blood Gas Analyzer (Radiometer Medical ApS, Bronshoj, Denmark). Coronary perfusion pressure (CPP) was calculated as the difference between decompression diastolic aortic and time-coincident right atrial pressure measured at the end of each minute of precordial compression. CPP during VF was defined as the difference between the mean aortic and mean right atrial pressures. Oxygen metabolism parameters, including oxygen delivery (DO_2_) and oxygen consumption (VO_2_), were calculated.

#### Myocardial energy metabolites

After the animals were killed at 24 hours after ROSC, the hearts were excised, and the right ventricles and both atria were removed. The myocardium was sampled from the anterior left ventricular wall starting at the apex and moving toward the base in a zigzag pattern to avoid vascular injury that could compromise blood supply to the region of the subsequent sample. Samples were immersed in liquid N_2_ within 10 seconds and then stored at −80°C and subsequently processed for ADP and ATP measurement using reversed-phase high-performance liquid chromatography (System Gold HPLC system with 32 Karat Software 5.0; Beckman Coulter, Brea, CA, USA) [17]. The percentage of phosphorylated creatine was calculated relative to the combined phosphocreatine and creatine pool. Enzyme activity was assessed by measuring the optical density of inorganic phosphate decomposed from ATP by the tissue protein according to the method of Isbir *et al*. [18]. Na^+^-K^+^-ATPase and Ca^2+^-ATPase enzyme activity was determined using standard formulas.

#### Biochemical parameter analysis in microdialysis

A CMA600 Microdialysis Analyser (CMA Microdialysis AB) was utilized for the quantitative determination of glucose, lactate, glutamate, pyruvate and glycerol in the ISF. All procedures were conducted in adherence to the manufacturer’s instructions using the original (manufacturer-supplied) reagent kits and calibrators. The analytic methods were based on enzymatic colorimetric assays [19]. All samples were collected in glass vials and sealed with Chromacol crimp caps (Thermo Scientific, Waltham, MA, USA) directly after sampling.

### Survival

The survival rate was determined based on the animals that survived the experimental protocol starting at ROSC until 24 hours after ROSC. Animals that died during surgical recovery were excluded.

### Statistical analysis

The results are expressed as mean ± SD. Student’s *t*-test was used for comparisons between every two groups. Differences at different time points were assessed by repeated-measures analysis of variance and the Bonferroni correction for *post hoc* comparisons between multiple experimental groups. In addition, the continuous variables were fixed to normal distributions and equal variances were analyzed by using the Kolmogorov-Smirnov test and homogeneity of variance test. Survival analysis was performed using the method of Kaplan and Meier, and comparisons between groups were made using the logrank test. A *P*-value <0.05 was considered statistically significant. All analyses were conducted using SPSS 17.0 software (SPSS, Chicago, IL, USA) and GraphPad Prism version 6 software (GraphPad Software, La Jolla, CA, USA).

## Results

### Baseline status

Baseline hemodynamic measurements and oxygen metabolism measurements are shown in Table 1. None of the variables (body weight, HR, MAP, MPAP, CO, lactate concentration, DO_2_, VO_2_ and extraction of oxygen differed significantly between the three groups (*P* > 0.05).

**Table 1:** **Baseline characteristics** ^**a**^

	**Sham group**	**SA group**	**Sildenafil group**
	**(** ***n*** **= 8)**	**(** ***n*** **= 8)**	**(** ***n*** **= 8)**
Weight, kg	29.13 ± 2.16	30.63 ± 0.92	30.38 ± 0.92
HR, beats/min	99.00 ± 7.44	101.38 ± 8.30	100.50 ± 10.04
MAP, mmHg	103.12 ± 5.19	101.88 ± 5.22	87.00 ± 5.81
MPAP, mmHg	23.42 ± 4.32	24.13 ± 5.24	24.34 ± 4.56
CO, L/min	2.86 ± 0.22	2.99 ± 0.20	2.99 ± 0.19
DO_2_, ml/min	424 ± 35	445 ± 34	450 ± 28
VO_2_, ml/min	112 ± 12	112 ± 9	115 ± 12
ERO_2_, %	24.17 ± 2.34	25.44 ± 2.70	25.46 ± 1.49
Lac, mmol/L	1.21 ± 0.49	1.31 ± 0.72	1.23 ± 0.01

^a^Values are mean ± SD. CO, Cardiac output; DO_2_, Oxygen delivery; ERO_2_, Extraction of oxygen; HR, Heart rate; Lac, Lactate; MAP, Mean aortic pressure; MPAP, Mean pulmonary arterial pressure; SA, Saline; VO_2_, Oxygen consumption.

### Resuscitation outcomes and survival

**Figure 3:**
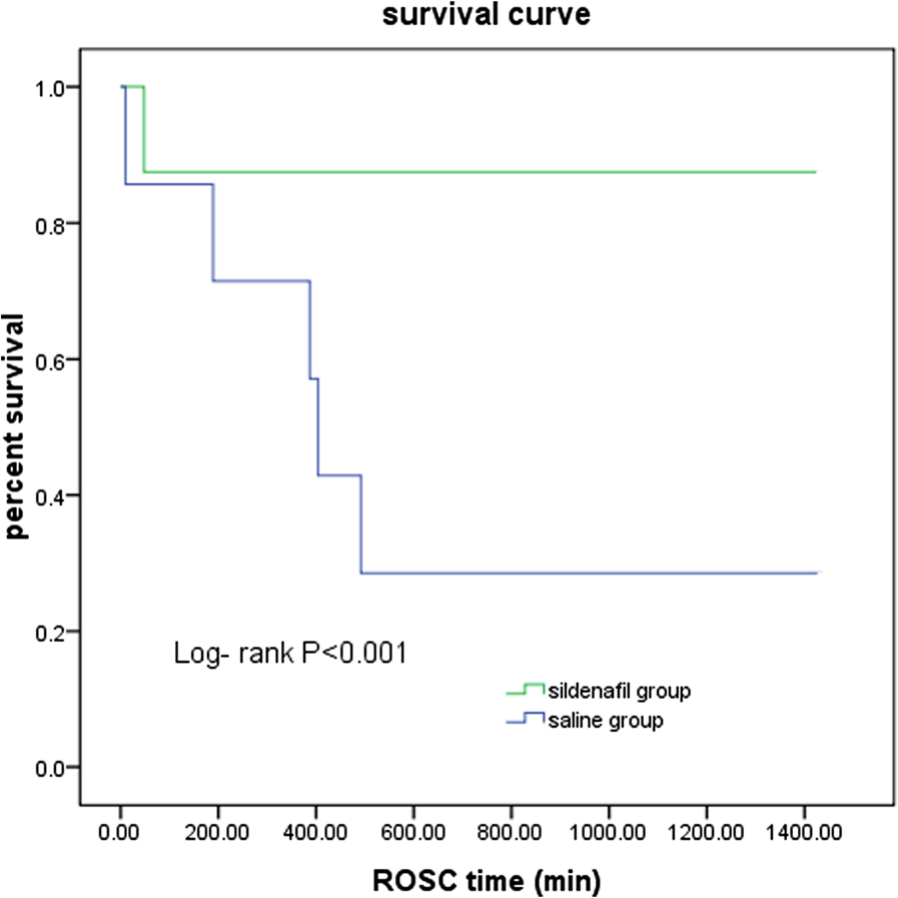
**Kaplan-Meier survival curve.** We found a significant difference in survival between the sildenafil group and the saline group (*P <* 0.001 by logrank test).

Resuscitation outcomes are shown in Table 2. None of the 16 animals achieved ROSC after initial defibrillation attempts. By comparison, the cumulative defibrillation energy was significantly lower in the sildenafil group than in the SA group (*P* < 0.0001). ROSC was achieved in all eight piglets in the sildenafil group and in seven of eight piglets in the SA group. There were no significant differences in 6-hour survival rate between the two groups (SA group and sildenafil group) (*P* = 0.83). In the SA group, four piglets died at 10 minutes, 189 minutes, 387 minutes and 404 minutes after ROSC, respectively. In the sildenafil group, only one piglet died at 48 minutes after ROSC. A significant difference in survival to the end of the 24-hour experiment period between the sildenafil and SA groups was demonstrated using the Kaplan-Meier survival curve and the logrank test (*P* < 0.05) (Figure 3).

**Table 2:** **Cardiopulmonary resuscitation outcomes** ^**a**^

	**SA group**	**Sildenafil group**	***P*** **-values**
	**(** ***n*** **= 8)**	**(** ***n*** **= 8)**	
Number of defibrillatory shocks	4.31 ± 1.62	2.91 ± 0.83	0.81
Cumulative defibrillation energy (J)	145.01 ± 33.41	95.02 ± 33.41	<0.001
Duration of CPR before ROSC (min)	6.12 ± 2.21	4.71 ± 1.32	0.25
6-hour survival	5	7	0.83
24-hour survival	3	7	0.04

^a^Values are mean ± SD or number (*n*). CPR, Cardiopulmonary resuscitation; ROSC, Restoration of spontaneous circulation; SA, Saline.

### Left ventricular function evaluation by invasive hemodynamic variables and oxygen metabolism status

**Figure 4:**
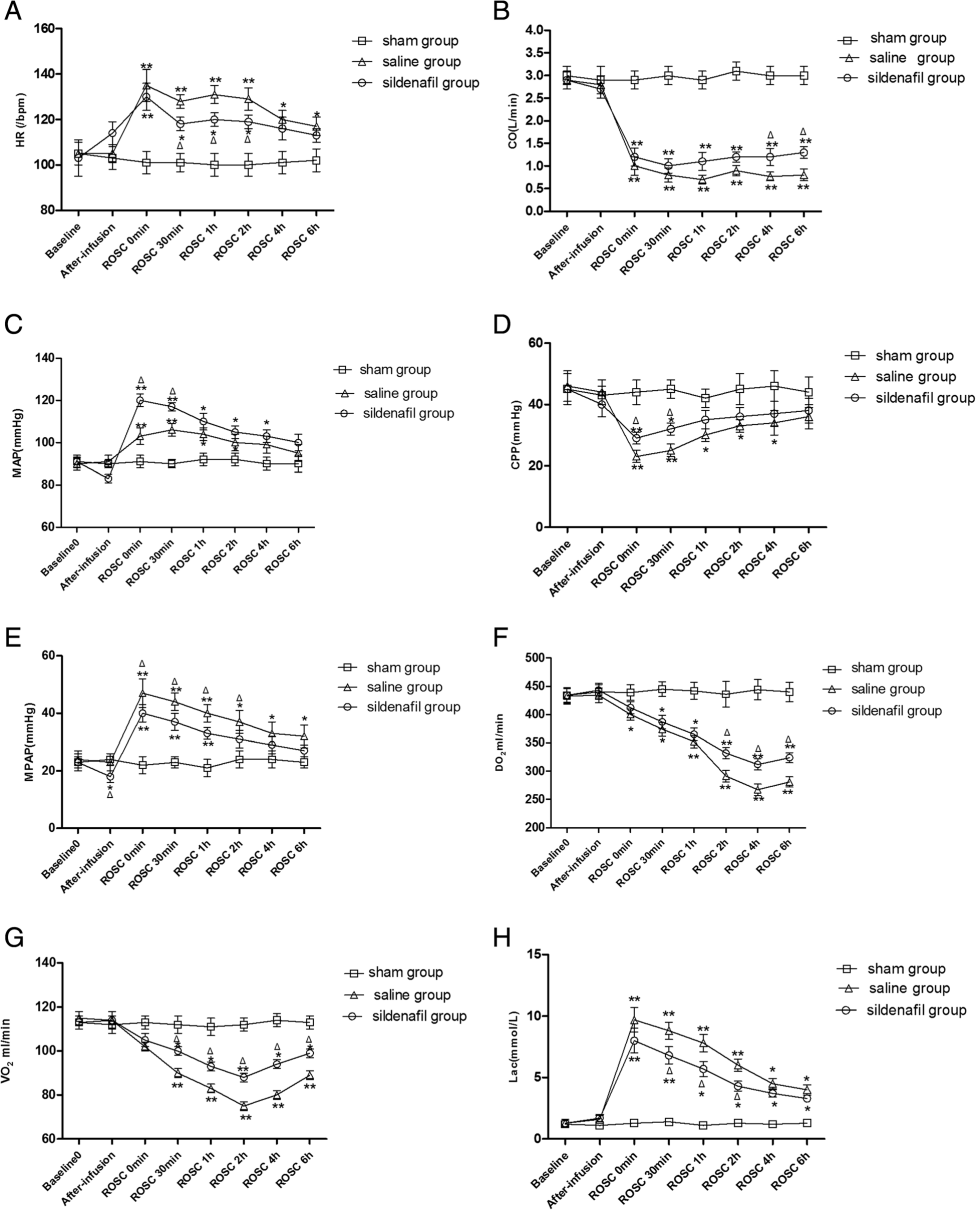
**Left ventricular function evaluation based on invasive hemodynamic variables and oxygen metabolism. (A)** Heart rate (HR). **(B)** Cardiac output (CO). **(C)** Mean aortic pressure (MAP). **(D)** Coronary perfusion pressure (CPP). **(E)** Mean pulmonary arterial pressure (MPAP). **(F)** Oxygen delivery (DO_2_). **(G)** Oxygen consumption (VO_2_). **(H)** Lactate (Lac). The values are reported as mean ± SD. ROSC, restoration of spontaneous circulation; SA, saline. *P < 0.05 vs. sham, **P < 0.01 vs. sham, ^Δ^ *P* < 0.05 vs. saline (one-way repeated-measures analysis of variance).

After ROSC, HR was significantly lower in the sildenafil group compared with the SA group at 1 and 2 hours after ROSC (*P* = 0.04, *P* = 0.03, respectively); however, there were no significant differences between the two groups at any other time points (Figure 4A). The CO values were significantly higher in the sildenafil group than in the SA group at 4 hours and 6 hours after ROSC (*P* = 0.02, *P* = 0.04, respectively) (Figure 4B). The MAP and CPP values were significantly increased in the sildenafil group compared with the SA group at baseline and 30 minutes after ROSC (*P* < 0.05 for both) (Figures 4C and 4D). These values were similar between the groups at other time points. Notably, MPAP was elevated in the sildenafil group and the SA group after CA, and the values of MPAP in the sildenafil group were continuously lower than in the SA group at most time points (*P* < 0.05, respectively) (Figure 4E). Oxygen metabolism measurements were compared among the three groups. DO_2_ was significantly higher in the sildenafil group than in the SA group at 2, 4 and 6 hours after ROSC (*P* = 0.03, *P* = 0.02 and *P* = 0.04, respectively), and VO_2_ was significantly higher in the sildenafil group than in the SA group at 30 minutes and 1, 2, 4 and 6 hours after ROSC (*P* = 0.02, *P* = 0.03, *P* = 0.007, *P* = 0.03 and *P* = 0.04, respectively) (Figures 4F and 4G). Serum lactate concentrations were significantly increased throughout the study time points after ROSC compared with baseline values in two groups (the SA and sildenafil groups) (*P* < 0.05) (Figure 4H). However, the lactate concentrations were lower in the sildenafil group than in the SA group at 30 minutes and 1 and 2 hours after ROSC (*P* = 0.02, *P* = 0.03, *P* = 0.01, respectively) (see Additional file 1: Table S1 for details).

### Myocardial energy effects of sildenafil at 24 hours after ROSC

Myocardial tissue analysis revealed a dramatic increase in the content of ATP, ADP and phosphocreatine in the sildenafil group compared with the SA group at 24 hours after ROSC (*P* = 0.03, *P* = 0.02, *P* = 0.02, respectively) (Table 3). The activities of left ventricle Na^+^-K^+^-ATPase and Ca^2+^-ATPase were significantly increased in the sildenafil group compared with the SA group at 24 hours after ROSC (*P* = 0.03, *P* = 0.04, respectively) (Table 3). All of these data are consistent with rapid development of intense myocardial ischemia.

**Table 3:** **The contents of ATP, ADP, phosphocreatine, Na** ^**+**^ **-K** ^**+**^ **-ATPase and Ca** ^**2+**^ **-ATPase activities in left ventricle tissue at 24 hours after successful resuscitation** ^**a**^

**Group**	**Phosphocreatine (nmol/mg protein)**	**ATP (nmol/mg protein)**	**ADP (nmol/mg protein)**	**Na** ^**+**^ **-K** ^**+**^ **-ATPase (U)**	**Ca** ^**2+**^ **-ATPase (U)**
Sham	33.36 ± 7.27	16.49 ± 1.67	1.51 ± 0.12	9.21 ± 1.45	10.96 ± 0.94
SA	19.71 ± 9.41**	10.49 ± 1.08**	0.91 ± 0.10**	4.97 ± 1.04**	3.65 ± 1.01**
Sildenafil	26.43 ± 4.66*^†^	12.3 ± 1.11*^†^	1.11 ± 0.21*^†^	6.89 ± 1.37*^†^	4.58 ± 1.43**^†^

^a^Values are mean ± SD. SA, Saline. *P < 0.05 vs. sham, **P < 0.01 vs. sham, ^†^ *P* < 0.05 vs. SA. All calculations were performed using one-way repeated-measures analysis of variance. All posttests were performed using the Bonferroni method. Myocardial tissue analysis revealed a dramatic increase in the content of ATP, ADP and phosphocreatine in the sildenafil group compared with the SA group at 24 hours after ROSC (*P* = 0.03, *P* = 0.02, *P* = 0.02, respectively). The activities of left ventricle Na^+^-K^+^-ATPase and Ca^2+^-ATPase were significantly increased in the sildenafil group compared with the SA group at 24 hours after ROSC (*P* = 0.03, *P* = 0.04, respectively).

### Effect of the sildenafil on myocardial energy metabolites by microdialysis

**Figure 5:**
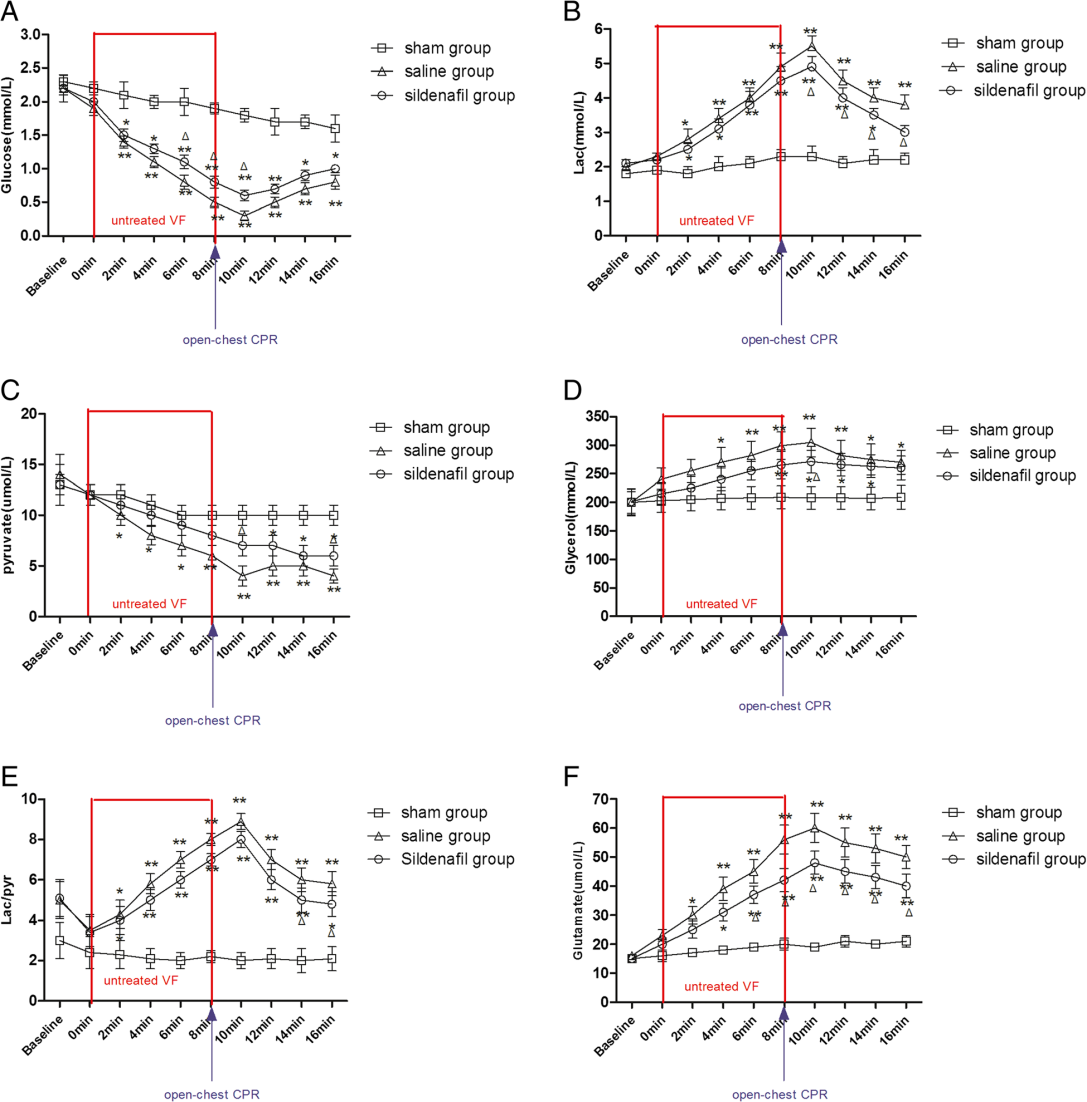
**The myocardial interstitial fluid concentrations of glucose, lactate, pyruvate, glycerol and glutamate as determined by microdialysis under basal conditions (before ventricular fibrillation) and after ventricular fibrillation. (A)** Myocardial interstitial fluid (ISF) concentrations of glucose. **(B)** ISF concentrations of lactate (Lac). **(C)** ISF concentrations of pyruvate. **(D):** ISF concentrations of glycerol. **(E)** ISF concentrations of lactate/pyruvate (Lac/pyr). **(F)** ISF concentrations of glutamate. The values are reported as mean ± SD. CPR, Cardiopulmonary resuscitation; ROSC, restoration of spontaneous circulation; VF, Ventricular fibrillation. *P < 0.05 vs. sham, **P < 0.01 vs. sham, ^Δ^ *P* < 0.05 vs. saline (one-way repeated-measures analysis of variance).

The microdialysis probes were implanted and extracted without inducing arrhythmias or other side effects. Real-time measurement of myocardial energy metabolic variables (glucose, pyruvate and glutamate), markers of injury (lactate and glycerol) and their ratios are presented in Figures 5A to 5E. No differences were noted at baseline between the groups. The mean ISF glucose levels decreased in all groups after CA and increased following CPR. The sildenafil group had a higher glucose level than the SA group during VF and the early reperfusion phase (*P* < 0.05) (Figure 5A). ISF lactate was elevated in all groups after VF, and the lactate levels rose further to reach a maximum after 10 minutes of VF and then declined slowly. Lactate levels in the SA group were continuously higher than in the other two groups (*P* < 0.05) (Figure 5B). The pyruvate levels were reduced significantly in the sildenafil and SA groups during VF; however, the levels of pyruvate were higher in the sildenafil group than in the SA group, even after CPR (Figure 5C). The glycerol level was elevated in the SA group after VF and stayed at a higher level compared with the other two groups, and the glycerol level of the sildenafil group also increased after VF and declined more significantly than in the SA group (Figure 5D). The lactate-to-pyruvate (L/P) ratios and glutamate levels in the SA group were dramatically higher than in the other two groups during and after CA (*P* < 0.05) (Figures 5E and 5F). Additional information is available in Additional file 1: Table S2.

## Discussion

We found that pretreatment with sildenafil reduced the severity of postresuscitation myocardial dysfunction during the no-flow or low-flow state of CA in this porcine model of CA. In our analysis of the changes of ISF metabolites (glucose, lactate, glutamate, pyruvate and glycerol) during VF and reperfusion, we observed that sildenafil inhibited abrupt increases in ISF lactate and glutamate levels, which indicates that sildenafil could improve myocardial energy metabolism during ischemia and reperfusion.

Although several studies have focused on the role of PDE5 inhibition on I/R injury in the myocardium [5-7], this study is, to our knowledge, the first to demonstrate that pretreatment with sildenafil prior to VF after CA is cardioprotective. Myocardial I/R injury occurs in a wide spectrum of patients, ranging from survivors of out-of-hospital CA to patients who have acute myocardial infarction and patients undergoing cardiac surgery. Ockaili *et al*. conducted the first study showing a powerful preconditioning-like effect of sildenafil against myocardial I/R injury in an *in vivo* rabbit model [20]. The anti-ischemic effects of sildenafil against I/R-triggered ventricular arrhythmias were also observed, as well as improvement of postischemic ventricular contractile function [21]. In another previous study, researchers demonstrated that, in an atherosclerotic mice model, sildenafil reduced oxidative stress and increased NO bioavailability, which culminated in protection against DNA damage [7]. All of these results are in accordance with our findings that the postresuscitation hemodynamic profile in the sildenafil group seemed to correspond with a less severe myocardial injury (higher ATP and Na^+-^K^+^-ATPase levels) and less dysfunction. This may be explained by an enhanced or better-preserved oxygen supply/demand ratio after successful ROSC in the sildenafil group (higher mean aortic pressure (MAP) and a lower HR/pressure radio), whereas the 24-hour survival rate showed a promising trend in the sildenafil group.

The magnitude of cardiac damage observed in piglets in the present study is similar to the damage observed in clinical practice (after CA and/or trauma). CA represents the most severe shock state, during which the delivery of oxygen and metabolic substrates is abruptly halted and metabolites are no longer removed [22]. Several studies have conclusively demonstrated that opening mitochondrial ATP-dependent potassium (mito-K_ATP_) channels plays an important role in ischemic as well as pharmacological preconditioning in the heart [23]. Opening of the mito-K_ATP_ channel partially compensates for the loss in membrane potential, which enables additional protons to be pumped out to form a H^+^ electrochemical gradient for both ATP synthesis and Ca^2+^ transport. In our present study, we found that the activities of Na^+^-K^+^-ATPase and Ca^2+^-ATPase were significantly increased in the sildenafil group compared with the SA group at 24 hours after ROSC, and we found that the contents of ATP, ADP and phosphodiesterase in the SA group were much lower than in the sildenafil group, indicating greater energy exhaustion in the SA group, which indicated that the myocardium energy metabolism system was damaged much more severely in the SA group than in the sildenafil group. We therefore suggest that sildenafil may lead to cardioprotection through beneficial effects on energy metabolism by opening mito-K_ATP_ channels. Our results are consistent with some previous experiments in which administration of sildenafil was shown to inhibit the breakdown of cGMP and subsequent high cGMP levels led to acute and delayed cardioprotection via mito-K_ATP_ channel opening and mediated preconditioning in rat hearts. These features would in principle lead to cardioprotection [20].

*In vivo* microdialysis techniques have made it possible to directly determine myocardial ISF metabolite changes. Therefore, in contrast to many previous studies focused only on serum markers, the findings of the present study provide comprehensive insight regarding metabolic changes and cellular energy preservation in ISF during CA by microdialysis.

The microdialysis results demonstrated real-time changes of several metabolic markers of basic metabolism in the ISF. It has been reported that the glucose level in the dialysate is closely correlated to the oxygen supply to the mycardium [24]. In the present study, after CA, glucose concentrations were significantly lower in the SA and sildenafil groups compared to the sham group (*P* < 0.05), whereas the glucose level remained higher in the sildenafil group than in the SA group during CA and in the early reperfusion phase. The reason for these effects is the shift of cellular metabolism to anaerobic conditions, which causes formation of lactate and an accelerated uptake of extracellular glucose during ischemia to sustain the energetically inefficient anaerobic glycolysis [25].

Lactate is produced by anaerobic glycolysis when the metabolic demand for oxygen exceeds the available oxygen supply [26]. Furthermore, lactate is the end product of anaerobic metabolism, and pyruvate is the intermediate product between aerobic and anaerobic metabolism. In aerobic conditions, pyruvate is produced via glycolysis and then enters the tricarboxylic acid cycle, largely bypassing the production of lactate [27]. In our study, the SA group had the highest lactate level and the lowest pyruvate level among the groups. Interestingly, compared with the SA group, sildenafil had almost a lower level of lactate and a higher level of pyruvate, suggesting that the sildenafil group had a potent capacity to clear metabolic waste accumulated in a relatively short CA duration.

The L/P ratio is a more reliable variable than lactate that characterizes the relationship between aerobic and anaerobic metabolism [28]. The animals in the SA group showed rapid deterioration of energy supply with an immediate increase of the L/P ratio as a sign of nutritional disorder. The L/P ratio in the sildenafil group was lower than that in the SA group at most time points, which demonstrated that animals treated with sildenafil had a better energy reservoir. Nevertheless, the curve of the L/P ratio for the sildenafil group suggested that sildenafil could minimize energy depletion to the utmost extent.

Increasing levels of glycerol indicated the degree of destruction in the cell membrane [29]. Sildenafil attenuated the production of glycerol at most time points, which indicates that it had a fair capability to protect myocardial cells.

There are obvious limitations to the design of this study. First, we acknowledge that our study may lack the power to detect some additional differences because of a relatively small sample size. Second, a major limitation is that the animals were administered sildenafil 30 minutes prior to VF, which cannot reflect sildenafil’s clinical applicability truly. Third, it also should be noted that the endpoints of this study were focused on the acute changes in postresuscitation myocardial dysfunction. The animals were killed immediately at the end of the study, resulting in a lack of observation of survival rate changes over a longer time course. An additional disadvantage of the study is the use of heparin to avoid hypercoagulability. Heparin may have impaired thrombus formation and vasoconstriction, which are physiologic responses in unanesthetized animals. Also, in the interpretation of our findings, repetitive electrical shocks themselves may increase the severity of postresuscitation myocardial dysfunction in settings of myocardial ischemia [30]. Further, because our studies were performed in an animal model in the absence of underlying cardiovascular disease, direct applicability to humans cannot be assured. Finally, optimal doses and methods of administration of sildenafil also deserve additional investigation. This information would be helpful in applying the use of sildenafil in CPR.

## Conclusions

On the basis of these experimental studies, we conclude that the administration of sildenafil increases the success of initial resuscitation and ameliorates myocardial dysfunction by improving myocardial metabolism. These findings suggest a critical protective role of sildenafil in postresuscitation myocardial dysfunction. This study provides a novel treatment target for protection against postresuscitation myocardial dysfunction.

## Key messages

- Compared with the saline group, the sildenafil group had better outcomes in terms of hemodynamic and oxygen metabolism parameters as well as 24-hour survival rate.
- Sildenafil could reduce the severity of postresuscitation myocardial dysfunction and interstitial fluid metabolite changes.
- This work might provide insights into the development of a novel strategy to treat postresuscitation myocardial dysfunction.

## Additional file

Additional file 1:
**Supplementary tables.** **Table S1.** Left ventricular function evaluation by invasive hemodynamic variables and oxygen metabolism status. **Table S2.** Effect of the sildenafil on myocardial energy metabolites by microdialysis.

## Abbreviations

CA: Cardiac arrest
cGMP: Cyclic guanosine monophosphate
CO: Cardiac output
CPP: Coronary perfusion pressure
CPR: Cardiopulmonary resuscitation
DO_2_: Oxygen delivery
ERO_2_: Extraction of oxygen
HR: Heart rate
I/R: Ischemia/reperfusion
ISF: Interstitial fluid
L/P: Lactate-to-pyruvate
MAP: Mean aortic pressure
MPAP: Mean pulmonary arterial pressure
NO: Nitrogen oxide
PDE-5: Isoform 5 of the enzyme phosphodiesterase
ROSC: Return of spontaneous circulation
SA: Saline
SHAM: Sham
VO_2_: Oxygen consumption

## References

[1] Nichol G, Thomas E, Callaway CW, Hedges J, Powell JL, Aufderheide TP, Rea T, Lowe R, Brown T, Dreyer J, Davis D, Idris A, Stiell, the Resuscitation Outcomes Consortium Investigators (2008). Regional variation in out-of-hospital cardiac arrest incidence and outcome. JAMA..

[2] Kern KB, Hilwig RW, Rhee KH, Berg RA (1996). Myocardial dysfunction following resuscitation from cardiac arrest: an example of global myocardial stunning. J Am Coll Cardiol.

[3] Neumar RW, Nolan JP, Adrie C, Aibiki M, Berg RA, Böttiger BW, Callaway C, Clark RS, Geocadin RG, Jauch EC, Kern KB, Laurent I, Longstreth WT, Merchant RM, Morley P, Morrison LJ, Nadkarni V, Peberdy MA, Rivers EP, Rodriguez-Nunez A, Sellke FW, Spaulding C, Sunde K, Vanden Hoek T (2008). Post–cardiac arrest syndrome: epidemiology, pathophysiology, treatment, and prognostication. A consensus statement from the International Liaison Committee on Resuscitation (American Heart Association, Australian and New Zealand Council on Resuscitation, European Resuscitation Council, Heart and Stroke Foundation of Canada, Inter-American Heart Foundation, Resuscitation Council of Asia, and the Resuscitation Council of Southern Africa); the American Heart Association Emergency Cardiovascular Care Committee; the Council on Cardiovascular Surgery and Anesthesia; the Council on Cardiopulmonary, Perioperative, and Critical Care; the Council on Clinical Cardiology; and the Stroke Council. Circulation.

[4] Choi DE, Jeong JY, Lim BJ, Chung S, Chang YK, Lee SJ, Na KR, Kim SY, Shin YT, Lee KW (2009). Pretreatment of sildenafil attenuates ischemia-reperfusion renal injury in rats. Am J Physiol Renal Physiol.

[5] Loganathan S, Radovits T, Hirschberg K, Korkmaz S, Barnucz E, Karck M, Szabó G (2008). Effects of selective phosphodiesterase-5-inhibition on myocardial contractility and reperfusion injury after heart transplantation. Transplantation.

[6] Elrod JW, Greer JJ, Lefer DJ (2007). Sildenafil-mediated acute cardioprotection is independent of the NO/cGMP pathway. Am J Physiol Heart Circ Physiol.

[7] Das S, Maulik N, Das DK, Kadowitz PJ, Bivalacqua TJ (2002). Cardioprotection with sildenafil, a selective inhibitor of cyclic 3′,5′-monophosphate-specific phosphodiesterase 5. Drugs Exp Clin Res.

[8] Guazzi M, Samaja M (2007). The role of PDE5-inhibitors in cardiopulmonary disorders: from basic evidence to clinical development. Curr Med Chem.

[9] Killingsworth CR, Wei CC, Dell’Italia LJ, Ardell JL, Kingsley MA, Smith WM, Ideker RE, Walcott GP (2004). Short-acting β-adrenergic antagonist esmolol given at reperfusion improves survival after prolonged ventricular fibrillation. Circulation.

[10] World Medical Association (2013). World Medical Association Declaration of Helsinki: Ethical Principles for Medical Research Involving Human Subjects. JAMA.

[11] Huang Q, Xu H, Yu Z, Gao P, Liu S (2010). Inbred Chinese Wuzhishan (WZS) minipig model for soybean glycinin and β-conglycinin allergy. J Agric Food Chem.

[12] Milano G, Bianciardi P, Rochemont V, Vassalli G, von Segesser LK, Corno AF, Guazzi M, Samaja M (2011). Phosphodiesterase-5 inhibition mimics intermittent reoxygenation and improves cardioprotection in the hypoxic myocardium. PLoS One.

[13] Zhang Q, Li C (2013). Combination of epinephrine with esmolol attenuates post-resuscitation myocardial dysfunction in a porcine model of cardiac arrest. PLoS One.

[14] Gilinsky MA, Faibushevish AA, Lunte CE (2001). Determination of myocardial norepinephrine in freely moving rats using in vivo microdialysis sampling and liquid chromatography with dual-electrode amperometric detection. J Pharm Biomed Anal.

[15] Zoerner F, Semenas E (2014). Resuscitation with amiodarone increases survival after hemorrhage and ventricular fibrillation in pigs. J Trauma Acute Care Surg.

[16] Wang S, Li C, Ji X, Yang L, Su Z, Wu J (2010). Effect of continuous compressions and 30:2 cardiopulmonary resuscitation on global ventilation/perfusion values during resuscitation in a porcine model. Crit Care Med.

[17] Ayoub IM, Kolarova JD, Kantola RL, Radhakrishnan J, Wang S, Gazmuri RJ (2007). Zoniporide preserves left ventricular compliance during ventricular fibrillation and minimizes postresuscitation myocardial dysfunction through benefits on energy metabolism. Crit Care Med.

[18] Isbir CS, Doğan R, Farsak B, Aydin M, Kilinç K (2000). The protective effect of lisinopril on membrane-bound enzymes in myocardial preservation. Cell Biochem Funct.

[19] Mantovani V, Kennergren C, Bugge M, Sala A, Lönnroth P, Berglin E (2010). Myocardial metabolism assessed by microdialysis: a prospective randomized study in on- and off-pump coronary bypass surgery. Int J Cardiol.

[20] Ockaili R, Salloum F, Hawkins J, Kukreja RC (2002). Sildenafil (Viagra) induces powerful cardioprotective effect via opening of mitochondrial K_ATP_ channels in rabbits. Am J Physiol Heart Circ Physiol.

[21] Bremer YA, Salloum F, Ockaili R, Chou E, Moskowitz WB, Kukreja RC (2005). Sildenafil citrate (Viagra) induces cardioprotective effects after ischemia/reperfusion injury in infant rabbits. Pediatr Res.

[22] Fryer RM, Hsu AK, Eells JT, Nagase H, Gross GJ (1999). Opioid-induced second window of cardioprotection: potential role of mitochondrial K_ATP_ channels. Circ Res.

[23] Gross GJ (2000). The role of mitochondrial K_ATP_ channels in cardioprotection. Cardiol.

[24] Bliss TM, Sapolsky RM (2001). Interactions among glucose, lactate and adenosine regulate energy substrate utilization in hippocampal cultures. Brain Res.

[25] Mizock BA, Falk JL (1992). Lactic acidosis in critical illness. Crit Care Med.

[26] Demers P, Elkouri S, Martineau R, Couturier A, Cartier R (2000). Outcome with high blood lactate levels during cardiopulmonary bypass in adult cardiac operation. Ann Thorac Surg.

[27] Polet F, Feron O (2013). Endothelial cell metabolism and tumour angiogenesis: glucose and glutamine as essential fuels and lactate as the driving force. J Intern Med.

[28] Liang MY, Tang ZX, Chen GX, Rong J, Yao JP, Chen Z, Wu ZK (2014). Is selective antegrade cerebral perfusion superior to retrograde cerebral perfusion for brain protection during deep hypothermic circulatory arrest? Metabolic evidence from microdialysis. Crit Care Med.

[29] Marklund N, Salci K, Lewén A, Hillered L (1997). Glycerol as a marker for post-traumatic membrane phospholipid degradation in rat brain. Neuroreport.

[30] Xie J, Weil MH, Sun S, Tang W, Sato Y, Jin X, Bisera J (1997). High-energy defibrillation increases the severity of postresuscitation myocardial dysfunction. Circulation.

