# Supplementary material for: The protective effects of a phosphodiesterase 5 inhibitor, sildenafil, on postresuscitation cardiac dysfunction of cardiac arrest: metabolic evidence from microdialysis
Source: Crit Care. 2014 Dec 5;18(6):641. doi: 10.1186/s13054-014-0641-7 (PMC4262990; doi:10.1186/s13054-014-0641-7)
Supplement: Additional file 1: — Supplementary tables. Table S1. Left ventricular function evaluation by invasive hemodynamic variables and oxygen metabolism status. Table S2. Effect of the sildenafil on myocardial energy metabolites by microdialysis. [file 13054_2014_641_MOESM1_ESM.doc]

**Additional file 1**

**Table S1:** Left ventricular function evaluation by invasive hemodynamic variables and oxygen metabolism status

|  |  | **P-values** | | | | | | |
| --- | --- | --- | --- | --- | --- | --- | --- | --- |
| **variables** |  | After-infusion | ROSC 0min | ROSC 30min | ROSC 1h | ROSC 2h | ROSC 4h | ROSC 6h |
| **HR (bpm)** | SA vs SHAM | 0.85 | <0.001 | <0.001 | <0.001 | <0.001 | 0.04 | 0.03 |
|  | Sildenafil *vs* SHAM | 0.78 | <0.001 | 0.03 | 0.03 | 0.02 | 0.04 | 0.21 |
|  | Sildenafil *vs* SA | 0.83 | 0.73 | 0.02 | 0.04 | 0.03 | 0.75 | 0.89 |
| **CO (L/min)** | SA *vs* SHAM | 0.91 | <0.001 | <0.001 | <0.001 | <0.001 | <0.001 | <0.001 |
|  | Sildenafil *vs* SHAM | 0.77 | <0.001 | <0.001 | <0.001 | <0.001 | <0.001 | <0.001 |
|  | Sildenafil *vs* SA | 0.89 | 0.23 | 0.31 | 0.45 | 0.33 | 0.02 | 0.04 |
| **MAP(mmHg)** | SA *vs* SHAM | 0.76 | <0.001 | <0.001 | 0.02 | 0.02 | 0.03 | 0.14 |
|  | Sildenafil *vs* SHAM | 0.91 | <0.001 | <0.001 | 0.02 | 0.03 | 0.19 | 0.28 |
|  | Sildenafil *vs* SA | 0.90 | 0.03 | 0.02 | 0.94 | 0.87 | 0.28 | 0.67 |
| **CPP(mmHg)** | SA *vs* SHAM | 0.73 | <0.001 | <0.001 | 0.03 | 0.03 | 0.02 | 0.21 |
|  | Sildenafil *vs* SHAM | 0.62 | <0.001 | 0.03 | 0.14 | 0.12 | 0.21 | 0.34 |
|  | Sildenafil *vs* SA | 0.71 | 0.03 | 0.04 | 0.39 | 0.24 | 0.34 | 0.76 |
| **MPAP(mmHg)** | SA *vs* SHAM | 0.61 | <0.001 | <0.001 | 0.003 | 0.03 | 0.04 | 0.04 |
|  | Sildenafil *vs* SHAM | 0.04 | <0.001 | 0.004 | 0.002 | 0.21 | 0.22 | 0.73 |
|  | Sildenafil *vs* SA | 0.04 | 0.04 | 0.03 | 0.03 | 0.02 | 0.77 | 0.81 |
| **DO_2_ (ml/min)** | SA *vs* SHAM | 0.21 | 0.03 | 0.02 | 0.007 | <0.001 | <0.001 | <0.001 |
|  | Sildenafil *vs* SHAM | 0.35 | 0.27 | 0.22 | 0.13 | 0.03 | 0.02 | 0.04 |
|  | Sildenafil *vs* SA | 0.45 | 0.56 | 0.24 | 0.22 | 0.03 | 0.02 | 0.04 |
| **VO_2_ (ml/min)** | SA *vs* SHAM | 0.72 | 0.12 | 0.007 | <0.001 | <0.001 | <0.001 | 0.006 |
|  | Sildenafil *vs* SHAM | 0.80 | 0.39 | 0.06 | 0.02 | 0.005 | 0.02 | 0.04 |
|  | Sildenafil *vs* SA | 0.63 | 0.81 | 0.02 | 0.03 | 0.007 | 0.03 | 0.04 |
| **Lac (mmol/L)** | SA *vs* SHAM | 0.52 | <0.001 | <0.001 | <0.001 | 0.006 | 0.04 | 0.03 |
|  | Sildenafil *vs* SHAM | 0.21 | <0.001 | 0.008 | 0.03 | 0.04 | 0.04 | 0.04 |
|  | Sildenafil *vs* SA | 0.34 | <0.001 | 0.02 | 0.03 | 0.01 | 0.88 | 0.34 |

Data in different group of each time point was compared with multivariate ANOVA. A value of p <0 .05

**Table S2: Effect of the sildenafil on myocardial energy metabolites by microdialysis**

|  |  | **P-values** | | | | | | | | |
| --- | --- | --- | --- | --- | --- | --- | --- | --- | --- | --- |
| **variables** |  | 0 min | 2 min | 4 min | 6 min | 8 min | 10 min | 12 min | 14 min | 16 min |
| **Glucose**  **(mmol/L)** | SA vs SHAM | 0.97 | 0.004 | 0.003 | <0.001 | <0.001 | <0.001 | <0.001 | 0.03 | 0.02 |
|  | Sildenafil *vs* SHAM | 0.89 | 0.03 | 0.02 | <0.001 | <0.001 | <0.001 | <0.001 | <0.001 | <0.001 |
|  | Sildenafil *vs* SA | 0.91 | 0.78 | 0.04 | 0.03 | 0.04 | 0.03 | 0.26 | 0.68 | 0.19 |
| **Lac**  **(mmol/L)** | SA *vs* SHAM | 0.76 | 0.03 | 0.006 | <0.001 | <0.001 | <0.001 | <0.001 | <0.001 | 0.007 |
|  | Sildenafil *vs* SHAM | 0.63 | 0.04 | 0.02 | <0.001 | <0.001 | <0.001 | <0.001 | 0.03 | 0.11 |
|  | Sildenafil *vs* SA | 0.85 | 0.66 | 0.24 | 0.73 | 0.42 | 0.04 | 0.03 | 0.03 | 0.02 |
| **Pyruvate**  **(umol/L)** | SA *vs* SHAM | 0.72 | 0.04 | 0.03 | 0.04 | <0.001 | <0.001 | <0.001 | <0.001 | <0.001 |
|  | Sildenafil *vs* SHAM | 0.76 | 0.14 | 0.24 | 0.12 | 0.28 | 0.28 | 0.03 | 0.04 | 0.03 |
|  | Sildenafil *vs* SA | 0.91 | 0.23 | 0.79 | 0.19 | 0.11 | 0.03 | 0.28 | 0.49 | 0.19 |
| **Glycerol**  **(mmol/L)** | SA *vs* SHAM | 0.34 | 0.25 | 0.03 | 0.006 | <0.001 | <0.001 | <0.001 | 0.02 | 0.03 |
|  | Sildenafil *vs* SHAM | 0.42 | 0.31 | 0.28 | 0.03 | <0.001 | 0.03 | 0.03 | 0.04 | 0.17 |
|  | Sildenafil *vs* SA | 0.83 | 0.74 | 0.62 | 0.29 | 0.31 | 0.04 | 0.32 | 0.61 | 0.28 |
| **Lac/pyr** | SA *vs* SHAM | 0.56 | 0.03 | 0.02 | <0.001 | <0.001 | <0.001 | <0.001 | <0.001 | <0.001 |
|  | Sildenafil *vs* SHAM | 0.49 | 0.04 | 0.03 | 0.007 | <0.001 | <0.001 | <0.001 | <0.001 | 0/02 |
|  | Sildenafil *vs* SA | 0.81 | 0.32 | 0.89 | 0.46 | 0.76 | 0.68 | 0.71 | 0.03 | 0.03 |
| **Glutamate**  **(umol/L)** | SA *vs* SHAM | 0.32 | 0.03 | 0.004 | <0.001 | <0.001 | <0.001 | <0.001 | <0.001 | <0.001 |
|  | Sildenafil *vs* SHAM | 0.45 | 0.12 | 0.007 | 0.008 | <0.001 | <0.001 | <0.001 | 0.006 | <0.001 |
|  | Sildenafil *vs* SA | 0.77 | 0.39 | 0.23 | 0.19 | 0.03 | 0.02 | 0.03 | 0.04 | 0.16 |

Data in different group of each time point was compared with multivariate ANOVA. A value of p <0 .05

was considered statistically significant.
